# A Tale of Two Afflictions: Transverse Myelitis and COVID-19 in a Young Female Patient

**DOI:** 10.7759/cureus.61066

**Published:** 2024-05-25

**Authors:** Vanessa Castellanos, Edwin R Mosquea Gomez, Emad Alatassi, David Blady, Bijal Mehta

**Affiliations:** 1 Internal Medicine, Hackensack Meridian Mountainside Medical Center, Montclair, USA; 2 Neurology, Hackensack Meridian Mountainside Medical Center, Montclair, USA

**Keywords:** viral transverse myelitis, covid 19 encephalitis, neuromyelitis optica spectrum disorder, acute transverse myelitis (atm), covid 19

## Abstract

Acute transverse myelitis (ATM) is a syndrome of multiple etiologies, with acute or subacute onset in which inflammation of the spinal cord results in neurological deficits, including weakness, sensory loss, and autonomic dysfunction. It is often associated with infectious or autoimmune etiologies but can be considered idiopathic when extensive workup is negative. We present a case of a young African American female who presented with acute onset of bilateral lower extremity weakness, loss of sensation, and autonomic dysfunction. On physical exam, she had absent lower extremity reflexes, 0-1/5 power, and markedly diminished sensation with no pain/temperature discrimination with an abdominal sensory level at T4. There was no upper extremity involvement. She was incidentally found to be COVID-19-positive and denied ever being vaccinated in the past. MRI of the spine revealed diffuse signal abnormality within the cervical and thoracic spine extending to the conus, and an MRI of the brain showed two white matter lesions in the frontal lobes. Lumbar puncture showed lymphocytic pleocytosis and elevated protein; Gram stain did not reveal any pathogen. The patient was treated initially with high doses of steroids with minimal response. She underwent multiple sessions of plasmapheresis with good tolerance and response. Differential diagnoses considered for this case were Guillain Barre syndrome, neuromyelitis optica (NMO), multiple sclerosis, SLE-induced transverse myelitis, or infectious cases. All lab work and workup came back negative for these diseases, leaving us with an interesting culprit: COVID-19 associated. There have been few cases mentioned in the literature of transverse myelitis caused by COVID-19, and this remains a possibility, as all other causes were ruled out.

## Introduction

Acute transverse myelitis (ATM) is a syndrome of multiple etiologies, with acute or subacute onset in which inflammation of the spinal cord results in neurological deficits, including weakness, sensory loss, and autonomic dysfunction. It is often associated with infectious or autoimmune etiologies but can be considered idiopathic when an extensive workup is negative [1]. We present a case of a young African American female who presented with acute onset of transverse myelitis in the setting of COVID-19-positive PCR.

## Case presentation

A 26-year-old female of African American descent, with a vague past medical history of bilateral lower extremity paresthesias two years ago that self-resolved, presented with acute onset of bilateral lower extremity weakness, loss of sensation, and autonomic dysfunction.

The patient reported nausea, vomiting, malaise, and subjective fevers, which later progressed within a few hours to severe lumbar back pain, rated 8/10 in intensity, with subsequent development of bilateral lower extremity weakness. She denied loss of sphincter control, vision changes, or a history of optic neuropathy. The patient had just returned from a cruise to Mexico one week before the presentation. She was exposed to street food, however, denied bathing in lakes, exploring caves, or exposure to sick contacts. She denied any fever, cough, dyspnea, dysuria, nausea, vomiting, or diarrhea while in Mexico. There was no relevant past family history. On physical exam, she had bilateral lower extremity 0-1/5 power proximal and distal weakness, absent patellar and Achilles reflexes bilaterally, and diminished sensation with no pain/temperature discrimination at an abdominal sensory level at T4. As for her upper extremities, there was no focal weakness noted, reflexes were intact, as well as a normal examination of cranial nerves.

**Figure 1:**
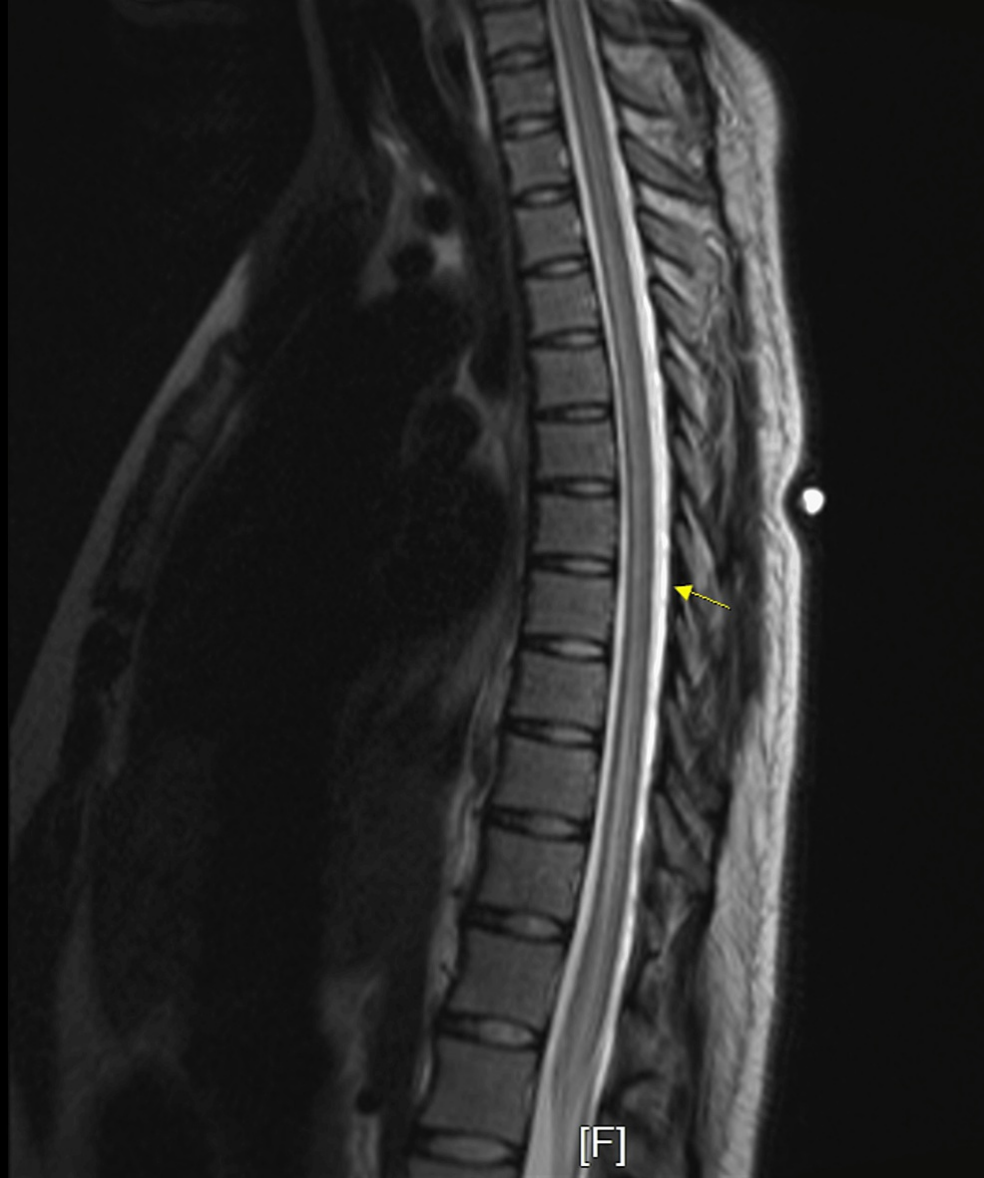
Magnetic resonance imaging (MRI) lumbar spine with and without contrast Diffuse signal abnormality seen within the cervical and thoracic cord, extending to the conus

**Figure 2:**
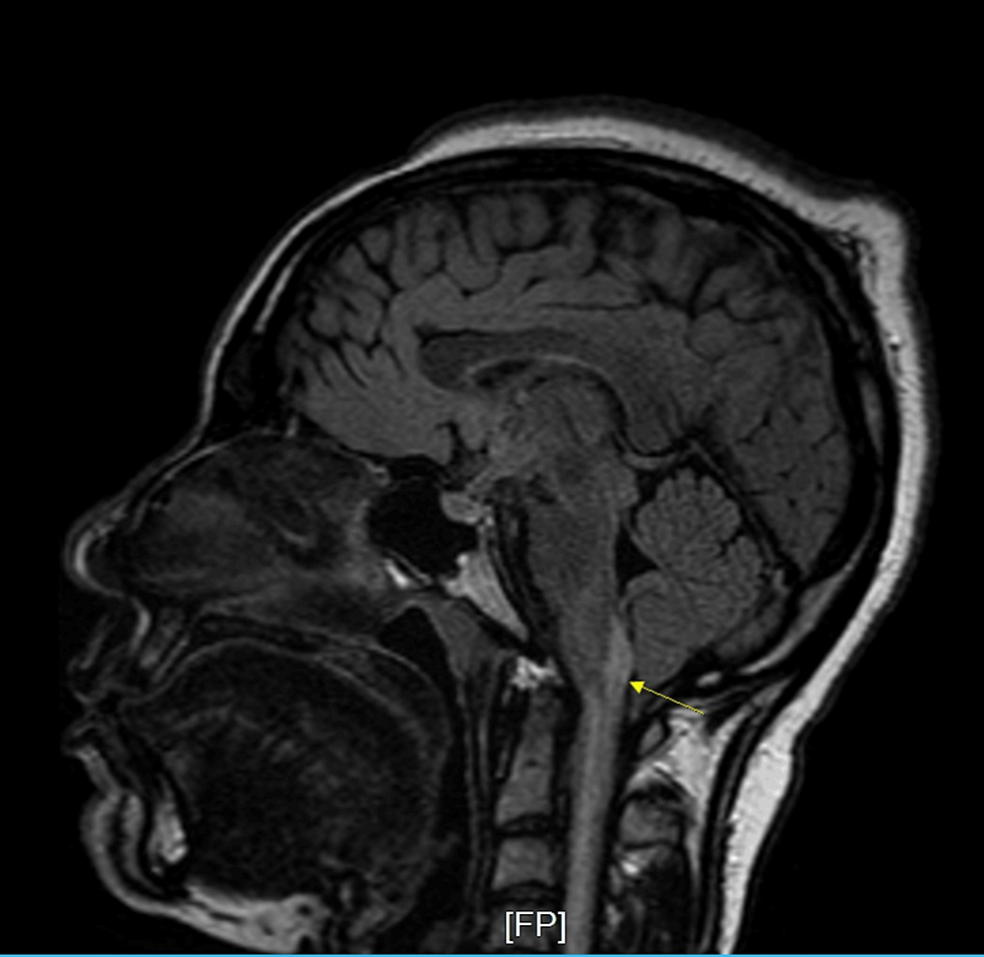
Magnetic resonance imaging (MRI) brain without contrast, sagittal FLAIR sequence Diffuse signal abnormality seen within the medulla extending into the visualized upper cervical cord seen to the C4 level FLAIR: fluid-attenuated inversion recovery

**Figure 3:**
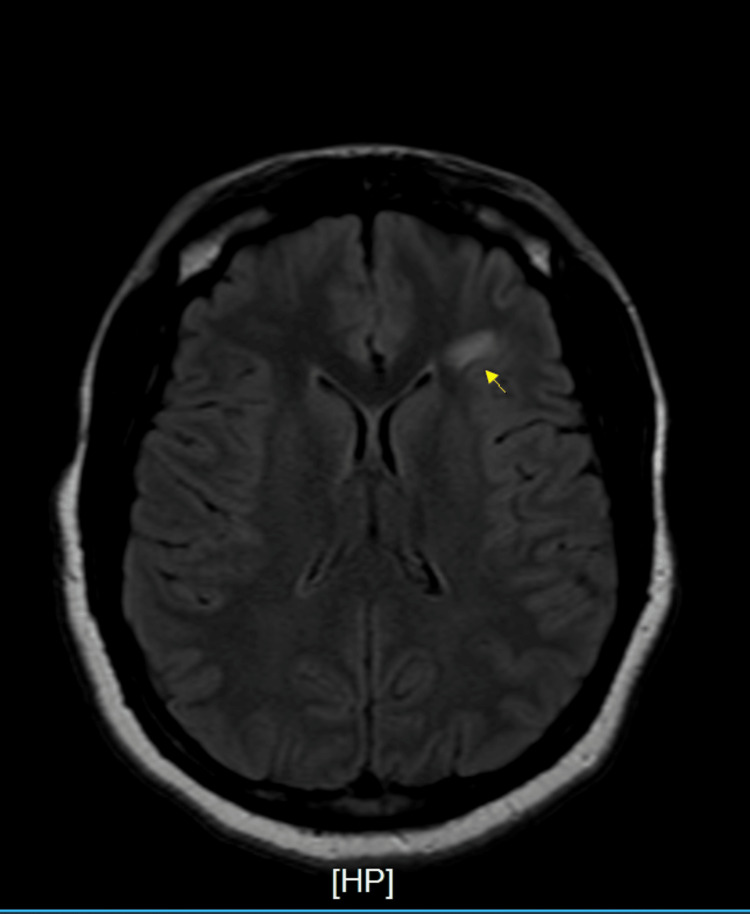
Magnetic resonance imaging (MRI) brain without contrast Larger area of subcortical FLAIR hyperintensity seen within the left frontal lobe, which has an oval configuration Fluid-attenuated inversion recovery

**Figure 4:**
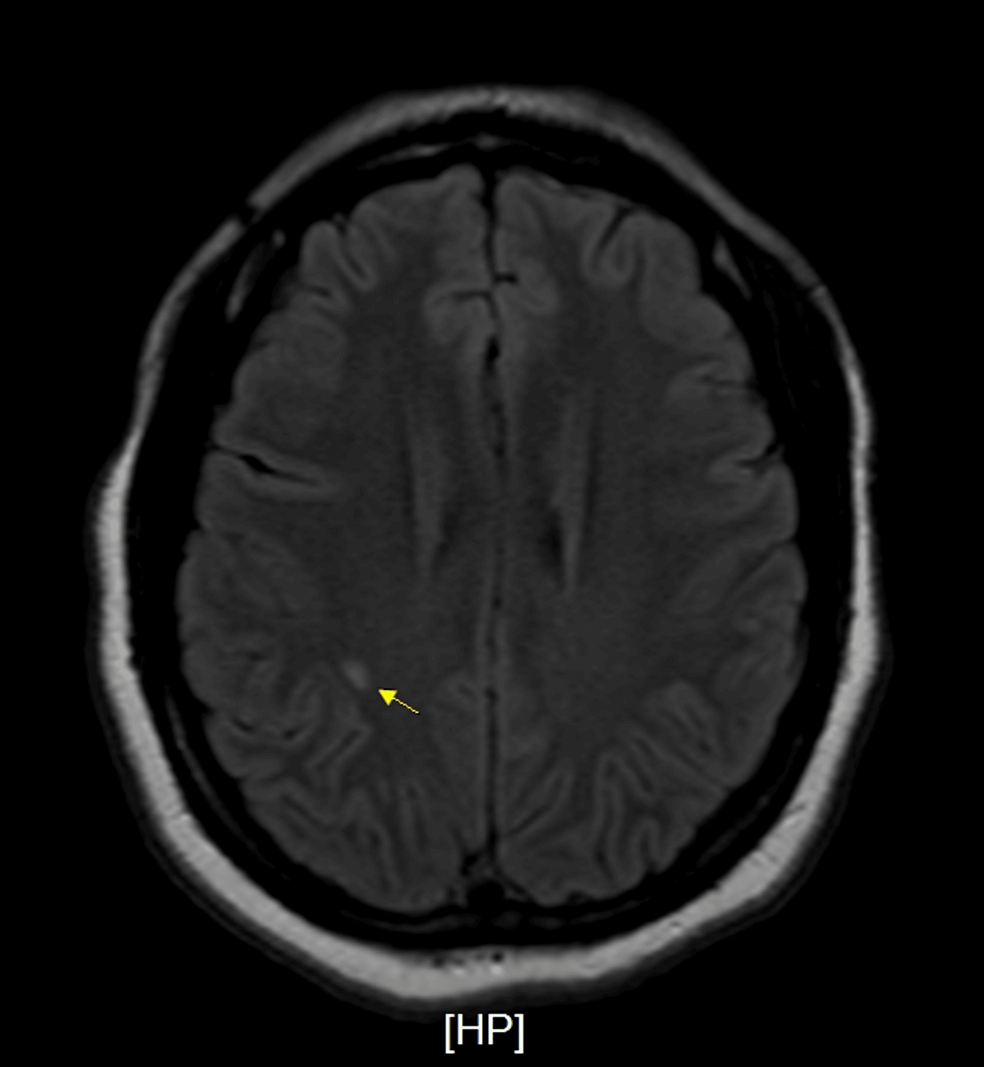
Magnetic resonance imaging (MRI) without contrast Focal area of oval shape signal abnormality seen within the subcortical white matter of the right posterior frontal lobe

Initial workup revealed COVID-19 positivity by PCR, an incidental finding, as the patient was asymptomatic. She denied any history of COVID-19 vaccination in the past. Chest X-ray was negative for infiltrates or effusions. CT scan of the head was negative for mass or hemorrhage. CT scan thoracic and lumbosacral spine showed a moderate diffuse disc bulge at L5-S1 and was negative for cord compression. MRI with and without contrast spine showed diffuse signal abnormality seen within the cervical and thoracic cord extending to the conus (Figure 1), as well as subtle abnormal intramedullary enhancement within the dorsal margin of the lower conus extending along the filum terminale. MRI head without contrast showed diffuse signal abnormality in the lower medulla (Figure 2) and visualized cervical cord with two white matter lesions in the frontotemporal lobes (Figure 3, 4).

The patient was admitted to the progressive care unit with careful monitoring of respiratory status. She was started on IV Solumedrol 250 mg every six hours for six days, with minimal to no improvement. A lumbar puncture was done and showed normal glucose, a lymphocytic pleocytosis, and elevated protein to 167. Immunoglobulin G (IgG) CSF was elevated to 20.4, CSF albumin was elevated to 93, and CSF culture and gram stain were negative. Zero oligoclonal bands were observed.

At this point, given the minimal improvement on high-dose steroids, the patient was started on plasmapheresis with a high suspicion of neuromyelitis optica (NMO). She received a total of 5 sessions of plasmapheresis over a total of 10 days. During a brief period (three days) the patient received remdesivir due to her COVID-19 positivity.

An extensive workup was done to rule out other potential causes for transverse myelitis such as autoimmune or infectious etiologies. ANA, anti-double-stranded DNA, Lyme antibody, RPR, HIV, and West Nile virus were negative, and the patient had normal levels of copper, vitamin E, vitamin B12, folate, and TSH. Urine drug screen was positive for cannabis, benzodiazepines, and amphetamines. NMO IgG/anti-Aquaporin 4 and MOG IgG were both sent to Mayo Clinic, which ultimately came back negative.

During her prolonged hospital stay the patient showed slow improvement in both her mobility and sensation in her lower extremities after treatment with plasmapheresis and physical therapy daily.

## Discussion

This is a rare case of a young female developing acute flaccid paralysis with sensory loss in bilateral lower extremities who was found to be incidentally COVID-19 positive. Initially, there was a high suspicion of neuromyelitis optica (NMO) due to several factors, including age, acute onset with bilateral involvement, MRI findings, as well as CSF analysis. The patient had clinical findings consistent with NMO even in the absence of optic neuritis and aquaporin antibody positivity [2].

Given the negative workup for other possible causes of transverse myelitis, we are left with a possible association between COVID-19 and the development of her condition. Coronavirus disease 2019 (COVID-19) has emerged as a virus mostly causing severe acute respiratory syndrome, but it has also been reported to cause neurological manifestations, possibly due to the invasion of the nervous system via the olfactory tract or the blood during the pro-inflammatory state [3,4].

Transverse myelitis has different causes, including autoimmune, post-infection, and post-vaccination as well as direct infection or even due to acquired demyelinating disease [3]. Almost 30-60% of idiopathic cases are reported to have an antecedent history of respiratory, gastrointestinal, or systemic disease [5]. Transverse myelitis has been associated with viral infections such as VZV and HSV-2, and now COVID-19 [6]. It is characterized by sensory, motor, and autonomic dysfunctions due to immune-mediated damage of the spinal cord [7]. Paraplegia, quadriplegia, loss of deep reflexes, sensory impairment, and bladder incontinence are some of the manifestations depending on an acute partial or complete dysfunction [3].

Diagnosing transverse myelitis due to COVID-19 can be challenging, making it a diagnosis of exclusion after all other common etiologies have been ruled out. MRI is the preferred imaging modality and can exclude other pathologies associated with cord lesions, showing a hyperintensity on T2-weighted images, with variable contrast enhancement [8]. Steroid therapy and plasmapheresis are the treatment modality of choice, with a resolution of symptoms in the vast majority of patients [9].

## Conclusions

Our patient presented with acute bilateral weakness, loss of reflexes and sensation, and neurogenic bladder. MRI findings showed hyperintensity in several lesions on her spinal cord as well as in an area of her brain, which could be seen in several diseases. However, given the negative workup for other causes, including Aquaporin-4 immunoglobin G, her clinical presentation of transverse myelitis was attributed to her COVID-19 positivity. Her presentation and response to treatment (high-dose steroids and plasmapheresis) are mostly consistent with possible COVID-19-associated transverse myelitis. However, it is important to mention that the possibility of seronegative neuromyelitis optica spectrum disorder (NMOSD) cannot be entirely excluded and should be reassessed in the future. There have been several case reports stating this association. Our goal is to continue to contribute to further expanding the knowledge on COVID-19, as there is yet much more to explore.
